# A Novel Medium for Isolating Two Japanese Species in the *Fusarium graminearum* Species Complex and a Dipstick DNA Chromatography Assay for Species Identification and Trichothecene Typing

**DOI:** 10.3390/jof8101048

**Published:** 2022-10-05

**Authors:** Haruhisa Suga, Masahiro Hayashi, Masayo Kushiro, Norichika Miyano, Hiroyoshi Inoue, Kaori Nakajima, Taku Kawakami, Takuji Tonooka, Takashi Nakajima, Masafumi Shimizu, Koji Kageyama

**Affiliations:** 1 Institute for Glyco-Core Research (iGCORE), Gifu University, Gifu 501-1193, Gifu, Japan; 2 The National Agriculture and Food Research Organization, 3-1-1 Kannondai, Tsukuba 305-8517, Ibaraki, Japan; 3 Miyagi Prefectural Furukawa Agricultural Experiment Station, 88 Fukoku, Ousaki, Furukawa 989-6227, Miyagi, Japan; 4 Mie Prefecture Agricultural Research Institute, 530 Ureshinokawakita, Matsusaka 515-2316, Mie, Japan; 5 Faculty of Applied Biological Sciences, Gifu University, Gifu 501-1193, Gifu, Japan; 6 River Basin Research Center, Gifu University, Gifu 501-1193, Gifu, Japan

**Keywords:** cereal disease, molecular diagnosis, plant pathogen, mycotoxin producer, multiplex PCR

## Abstract

Members of the *Fusarium graminearum* species complex (*Fg* complex) are the primary pathogens that cause Fusarium head blight in wheat and barley. *Fg* complex members grow poorly on *Fusarium oxysporum*-selective media, such as Komada and Fo-G2, that have also been used for the isolation of other *Fusarium* species. Therefore, Komada medium was modified as FG medium for the isolation of *Fg* complex members. However, the production of pentachloronitrobenzene that is the most effective component of FG medium is discontinued and new media is required for the selective isolation of *Fg* complex members. In addition, the rapid diagnosis of isolated fungi is useful for the disease control. Novel tools have been developed for isolating and characterizing *Fg* complex members. FG21, a semi-selective medium for isolating *Fg* complex members, was developed using potato dextrose agar. Furthermore, a dipstick DNA chromatography assay was developed both to identify *Fusarium graminearum* sensu stricto and *Fusarium asiaticum* in the *Fg* complex and their trichothecene mycotoxin types. The easier isolation and characterization of *Fg* complex members in Japan was attained by the combined use of FG21 medium and the dipstick DNA chromatography assay.

## 1. Introduction

Fusarium head blight (FHB) is a devastating disease affecting cereal crops. It reduces yield, quality, contaminates the grain with mycotoxins, and affects the overall production of wheat and barley worldwide. In North America, the total loss of wheat by FHB during 1991–1997 was estimated to be USD 1.3 billion [1]. FHB causes the premature bleaching of part or the entire wheat ear. In some cases, salmon-pink sporodochia can be observed at glume edges. Flowering wheat heads are susceptible to airborne spore infection. Precipitation during the flowering stage of wheat promotes an FHB outbreak. FHB pathogens include *Microdochium nivale* and *Fusarium* spp., such as members of the *Fusarium graminearum* species complex (*Fg* complex), *Fusarium culmorum*, *Fusarium avenaceum*, *Fusarium poae*, and *Fusarium pseudograminearum*.

Mycotoxins produced by FHB pathogens cause further problems in the harvested grains. Trichothecene mycotoxins are produced by *Fusarium* spp. but not *Microdochium nivale*. Members of the *Fg* complex are the primary pathogens of FHB and are distributed worldwide. Phylogenetic analyses revealed that the *Fg* complex consists of at least 16 distinct species [2,3,4,5,6]. In addition to wheat and barley, these members can impact other grains. Members of the *Fg* complex have also been known to be the pathogens responsible for Gibberella ear rot in maize; *Fusarium boothii*, an *Fg* species complex member, was markedly associated with this disease in South Africa [7]. *Fusarium asiaticum* has a host preference for rice, suggested by its higher propensity for perithecial formation on rice straw, when compared to *Fusarium graminearum* sensu stricto (s. str.) and *F. boothii* [8]. In Japan, *F. graminearum* s. str., *F. asiaticum*, and *Fusarium vorosii* have been identified as members of the *Fg* complex, although *F. vorosii* has rarely been isolated [3,9].

Members of the *Fg* complex are known to produce mycotoxins, namely zearalenone and trichothecenes. Trichothecenes inhibit peptidyltransferase activity by binding to the 60S ribosomal subunit [10]. The strains of trichothecene-producing *Fusarium* spp. have been classified into three types based on the trichothecene produced: nivalenol (NIV), 3-acetyl deoxynivalenol (3ADON), and 15-acetyl deoxynivalenol (15ADON). Previous investigations have revealed that species and trichothecene-type compositions of the *Fg* complex vary geographically. The NIV type of *F. graminearum* s. str. has been detected in the United States [11], whereas no NIV type has been found in 50 strains of *F. graminearum* s. str. in Japan [9]. However, in the case of Japanese *F. asiaticum*, 173 (70%) out of 246 strains were of the NIV type [9].

Polymerase chain reaction (PCR) methods have previously been developed to identify the species and trichothecene types (NIV, 3ADON, or 15ADON) of the *Fg* complex. Ward et al. [12] developed a multilocus genotyping assay, using multiplex PCR to identify 11 species and 3 trichothecene types. This assay produced a comprehensive and accurate assessment of *Fg* complex members using DNA polymorphisms, but it required allele-specific primer extension reactions, fluorescent microspheres, and equipment for signal detection. Starkey et al. [3] developed a multiplex PCR assay targeting *TRI3*, encoding the trichothecene 15-O-acetyltransferase, and *TRI12*, encoding trichothecene efflux pump, for trichothecene typing. Suga et al. [9] developed PCR-RFLPs to identify *F. graminearum* s. str. and *F. asiaticum*, based on a species-specific single-nucleotide polymorphism in the *histone H3* gene. Suzuki et al. [13] developed a multiplex PCR assay for the simultaneous identification of the species and trichothecene types in *F. asiaticum* and *F. graminearum* s. str. However, PCR-based methods generally require agarose gel electrophoresis to detect species-identifying amplicons. In contrast, the dipstick DNA chromatography assay is an alternative, quick, and simple detection method for PCR amplicons [14,15].

A selective medium, on which only specific microorganisms can grow, is critical for both microorganism detection and identification. Both Komada and Fo-G2 media have been developed to be *Fusarium oxysporum*-selective [16,17]. Komada medium has also been used for the isolation of other *Fusarium* spp., such as members of the *Fusarium solani* and *Fusarium incarnatum*–*equiseti* species complexes [18,19]. However, Komada medium cannot be used to isolate *Fg* complex members because they grow poorly on this medium [20]. In addition, Fo-G2 medium contains Befran (iminoctadine triacetate), which has been employed for FHB control in Japan, making this medium’s applicability to *Fg* complex unlikely due to its toxicity [21]. Togawa [20] modified the Komada medium for *Fg* complex members, naming it FG medium. One of the most effective components in the Komada and FG media for the selective growth of *Fusarium* spp. is pentachloronitrobenzene (PCNB). However, PCNB production is discontinued because it has not been a registered pesticide in Japan since 2000. Therefore, new media without PCNB is required for the selective isolation of *Fg* complex members.

In this study, the authors developed a semi-selective FG21 medium without PCNB for isolating *Fg* complex members and used a dipstick DNA chromatography assay to identify two species in the *Fg* complex and their trichothecene types. The amplicons in the multiplex PCR assay were visually detected on the dipstick within 20 min. Thus, the authors used this simpler method to isolate and characterize *Fg* complex members with both FG21 medium and dipstick DNA chromatography.

## 2. Materials and Methods

### 2.1. Media

FG21 medium was prepared as follows: potato dextrose broth (PDA; Difco Laboratories, Detroit, MI, USA) with 2% agar was autoclaved. Before PDA solidification, fungicide, antibiotics, and detergents were mixed; 0.015% (*w*/*v*) Rizolex wettable powder (Sumitomo Chemical Co., Ltd., Takarazuka, Japan), 0.01% (*w*/*v*) Trifumin wettable powder (Nippon Soda Co. Ltd., Tokyo, Japan), 0.125% (*w*/*v*) chloramphenicol, 0.05% (*v*/*v*) tergitol type NP-10, and 0.1% (*w*/*v*) sodium cholate were added. The prototype FG21 medium had 0.025% (*w*/*v*) chloramphenicol and lacked Trifumin but had the same composition as the final FG21 medium.

Oatmeal agar (Difco Laboratories) and cornmeal agar (Nissui, Tokyo, Japan) were prepared according to the manufacturers’ instructions. Sabouraud dextrose broth agar (Biokar Diagnostics, Beauvais, France) was prepared, as according to the manufacturer’s instructions, with addition of 2% (*w*/*v*) agar. Malt extract agar contained 2% (*w*/*v*) malt extract powder (Nacalai Tesque, Inc., Kyoto, Japan), 2% (*w*/*v*) D-(+)-glucose, 1% (*w*/*v*) Bacto Peptone (Difco Laboratories), and 2% (*w*/*v*) agar.

### 2.2. Strains and Growth Test

The strain information is summarized in Table 1. Strains of all *Fg* complex members and trichothecene types (*F. graminearum* s. str./3ADON and 15ADON, *F. asiaticum*/NIV, 3ADON, and 15ADON, and *F. vorosii*/15ADON) that have been detected in Japan [3,9] were used as the fungal strains to develop the FG21 medium and dipstick DNA chromatography assay. Other strains, including *Fusarium* spp., were used as reference fungi. Strains F1904008 and E1904019 were isolated from wheat seeds using prototype FG21 medium. Strains P1904014 and A1904015 were isolated from barley seeds. In these four strains, the internal transcribed spacer region nucleotide sequences of the nuclear ribosomal DNA were obtained by directly sequencing the PCR amplicons using the primer pair ITS1/ITS4 [22]. These results were used in a homology search at GenBank database for genus identification. The nucleotide sequences were deposited in the DDBJ/EMBL/GenBank database under the accession numbers LC719979-LC719982.

For the medium growth test, strains precultured on PDA at 25 °C for 1 week were cut using a sterile cork borer with a 4 mm diameter. Four strains were transplanted onto the medium in a 90 mm Petri dish and incubated at 25 °C for 4 days.

### 2.3. Fungal Isolation from Plant Source

Seeds or wheat heads were sterilized by sequential washing with 70% ethanol, 1% sodium hypochlorite containing 0.02% Tween-20, and sterile distilled water. After removing the water, samples were placed on FG21 medium in a 90 mm Petri dish. Reddish colonies formed on the medium around the plant materials in 3 d at 25 °C. If no reddish colonies appeared, the Petri dish was turned over, removing the plant material, and incubated further for several days. Aerial mycelia from each reddish colony were collected and used for DNA extraction. For colony purification, colonies were subcultured on synthetic nutrient agar (SNA) under black light for sporulation. The spore suspension collected from the culture on SNA was spread onto PDA, and a single colony was used for DNA extraction.

### 2.4. DNA Extraction

Simple and rapid DNA extraction was performed using the Kaneka Easy DNA Extraction Kit, version 2 (Kaneka, Tokyo, Japan), according to the manufacturer’s instructions with the following modifications: a crumb of a colony’s aerial mycelia was sniped with tweezers and transferred to a microtube containing two small metal screws and 100 μL of Reagent A. The mycelia crumb was crushed by vortexing for several minutes. Fourteen microliters of Reagent B was added, and the metal screws were removed with a micropipette tip. After centrifugation at 17,800× *g* for 1 min, the supernatant was used as the DNA solution for multiplex PCR.

### 2.5. Primer Design and Multiplex PCR Conditions

The primers used are listed in Table 2 (Tohoku Bio-Array, Sendai, Japan). The HS976/HS1039 primer pair was designed for *FGSG_17538*, which encodes an aldehyde dehydrogenase that is present only in the genome of *F. graminearum* s. str. among the genomes of *F. asiaticum*, *F. cortaderiae*, and *F. graminearum* s. str. [26]. The HS974/HS1035 primer pair was designed for *APS1*, which encodes a non-ribosomal peptide synthetase involved in apicidin biosynthesis and was present only in the genome of *F. asiaticum*, among the three genomes [26]. Starkey et al. [3] designed 3CON, 3NA, 3D15A, and 3D3A primers on *Tri3* for trichothecene typing using multiplex PCR. These primers were used in the current study, although the 5ʹ termini of forward primers were labelled with five different tag-linker sequences and reverse primers labelled with biotin (Tohoku Bio-Array).

Multiplex PCR for species identification was performed in 10 μL of a reaction mixture, containing 1 μL of 10× buffer, 1 μL of 2 mM dNTP mixture, 0.6 μL of 25 mM MgSO_4_, 2.0 μL of primer mix, 0.25 μL of 1 U/μL KOD-Plus-Neo DNA polymerase (Toyobo, Osaka, Japan), 4.15 μL of ultrapure water, and 1 μL of genomic DNA solution. Primer mix included 1.67 μM of HS970 and HS1039 and 0.83 μM of HS974 and HS1035. PCR was conducted using the T100 thermal cycler (Biorad, Hercules, CA, USA) under the following conditions: 94 °C for 2 min, 25 cycles of 94 °C for 30 s, and 66 °C for 30 s. Multiplex PCR for trichothecene typing was similarly performed, except with 3.2 μL of primer mix and 2.95 μL of ultrapure water. The primer mix included 1.25 μM of 3CON, 3D3A, 3D15A, and 2.5 μM of 3NA. A TaKaRa PCR Thermal Cycler TP600 (Takara, Otsu, Japan) was also used, with the cycle number increased to 30. For trichothecene typing, the multiplex PCR solution had 2.95 μL of ultrapure water replaced with primer mix.

Nucleotide sequences of PCR amplification region in *FGSG_17538* and *APS1* were used to search for homologous sequence in the Genbank genome database.

### 2.6. Dipstick DNA Chromatography Assay

The PCR amplicons were detected by dipstick DNA chromatography. For species identification, 5 μL of multiplex PCR solution was mixed with 5 μL of dipstick developing buffer and 1.5 μL of streptavidin-coated blue latex beads (Tohoku Bio-Array). The amplicons were bound to blue latex beads for 5 min by streptavidin-biotin association. The PCR solution for trichothecene typing was similarly prepared, then mixed with the species identification solution in a microtube. A dipstick (Tohoku Bio-Array) was inserted and left for 20 min for chromatography. The amplicon-tagged sequence hybridized to its corresponding anti-tag on the dipstick, and the positive reactions appeared as blue lines.

## 3. Results

### 3.1. Development of the FG21 Medium

*Fg* complex members are known to grow relatively fast on PDA, which is a general medium for fungal culturing. Initially, the authors prepared a prototype FG21 medium based on PDA, noting that red pigmentation appearing on this medium, which can be an indicator of *Fg* complex members. *F. graminearum* s. str., *F. asiaticum*, and *F. vorosii* grew on the prototype FG21 medium, yet they grew more slowly than on PDA. Prototype FG21 medium effectiveness was evaluated by an isolation trial of *Fg* complex members from wheat and barley seeds. Several reddish colonies appeared, but alongside many other fungal colonies. Four strains (F1904008, E1904019, P1904014, and A1904015) that were not *Fg* complex members based on colony appearance were obtained as references for evaluating the final FG21 medium.

The same components of prototype FG21 were mixed at the same concentrations in oatmeal agar, cornmeal agar, Sabouraud dextrose broth agar, and malt extract agar. However, red pigmentation in colonies was not observed on any of the media.

**Figure 1:**
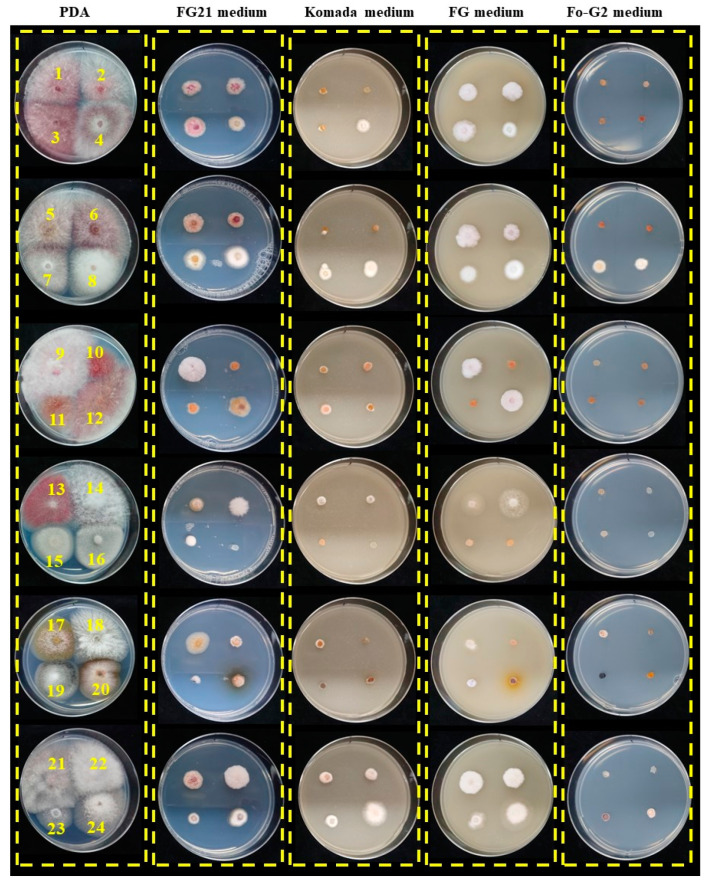
Fungal growth comparison of the FG21 medium and previously developed media. Potato dextrose agar (PDA) is a general medium for fungal culture and is used as the foundation of FG21 medium. Komada [16] and Fo-G2 [17] are *Fusarium oxysporum* selective media. FG is a *Fusarium graminearum*-selective medium [20]. The strains were cultured at 25 °C for 4 d. *Fusarium graminearum* sensu stricto Fg0101020 (1), Fg0201201 (2), *Fusarium asiaticum* Fa0244004 (3), Fa0239005 (4), *Fusarium vorosii* Fv0301112 (5), Fv0301831 (6), *Fusarium avenaceum* MAFF101042 (7), MAFF235547 (8), *Fusarium culmorum* MAFF101144 (9), NRRL3288 (10), *Fusarium cerealis* NRRL25805 (11), NRRL13721 (12), *Fusarium poae* MAFF236500 (13), MAFF236648 (14), *Microdochium nivale* MAFF235834 (15), MAFF236681 (16), *Fusarium* sp. F1904008 (17), *Pestalotiopsis* sp. P1904014 (18), *Alternaria* sp. A1904015. (19), *Epicoccum* sp. E1904019 (20), *Fusarium sporotrichioides* MAFF236638 (21), MAFF236639 (22), *Fusarium fujikuroi* Gfc0801001 (23), and Gfc0825009 (24).

To improve medium selectivity, Trifumin wettable powder was added to the prototype FG21 medium at 0.01% (*w*/*v*), 0.1%, or 1%. *F. graminearum* s. str., *F. asiaticum*, and *F. vorosii* showed no change in growth with 0.01% Trifumin, and growth reduction occurred with 0.1% and 1% Trifumin. Additionally, bacterial colonies were observed in the trial isolation of *Fg* complex members from wheat seeds using the prototype FG21 medium containing 0.01% Trifumin. These colonies were isolated, and their growth inhibition was measured using a range of chloramphenicol concentrations from 0.025% (*w*/*v*) to 0.125% in the prototype FG21 medium. Finally, the FG21 medium was established by adding 0.01% Trifumin and increasing the chloramphenicol concentration to 0.125%. To test its efficacy in isolating *Fg* complex members, the FG21 medium was compared with Komada, FG, and Fo-G2 media in a growth test using both *Fg* complex members and the reference fungal strains (Figure 1). *F. graminearum* s. str., *F. asiaticum*, and *F. vorosii* did not grow on Komada and Fo-G2 media; however, *F. asiaticum* Fa0239005 did grow on the Komada medium. On the prototype FG21 and final FG21 medium, all strains grew similarly, including *Fg* complex members, except *Fusarium* sp. F1904008.

### 3.2. Development of the Dipstick DNA Chromatography Assay

**Figure 2:**
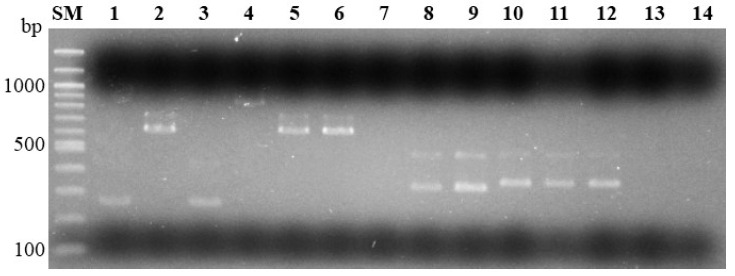
Multiplex PCR for species identification and trichothecene typing of the *Fusarium graminearum* species complex. Multiplex PCR with the primers 3CON, 3D3A, 3D15A, and 3NA for trichothecene typing. Expected amplicon sizes were 3-acetyl deoxynivalenol (3ADON): 243 bp; 15-acetyl deoxynivalenol (15ADON): 610 bp; and nivalenol (NIV): 840 bp. Multiplex PCR with primers HS974, HS976, HS1035, and HS1039 for species identification. Expected amplicon sizes for *Fusarium graminearum* sensu stricto (s. str.): 317 bp; *Fusarium asiaticum*: 332 bp. PCR amplicons were subjected to 1% agarose gel electrophoresis. Lane SM, 100 bp-size marker; lanes 1 to 7, multiplex PCR for trichothecene typing (lane 1, Fg0101020/3ADON; lane 2, Fg0201201/15ADON; lane 3, Fa0244004/3ADON; lane 4, Fa0239005/NIV; lane 5, Fa0343747/15ADON; lane 6, Fv0301112/15ADON; lane 7, water control); lanes 8 to 14, multiplex PCR for species identification (lane 8, Fg0101020/*F. graminearum* s. str.; lane 9, Fg0201201/*F. graminearum* s. str.; lane 10, Fa0244004/*F. asiaticum*; lane 11, Fa0239005/*F. asiaticum*; lane 12, Fa0343747/*F. asiaticum*; lane 13, Fv0301112/*Fusarium vorosii*; lane 14, water control).

The optimal multiplex PCR conditions for trichothecene typing and species identification were determined based on the specific target DNA amplification in all *Fg* complex members and trichothecene types that have been detected in Japan: *F. graminearum* s. str./3ADON and 15ADON; *F. asiaticum*/NIV, 3ADON, 15ADON; *F. vorosii*/15ADON (Figure 2).

**Figure 3:**
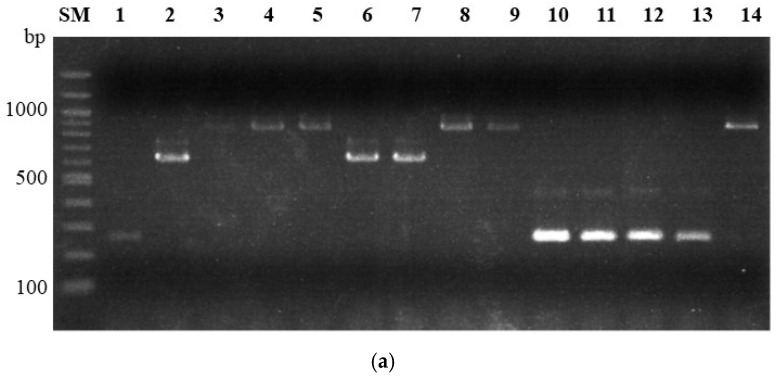

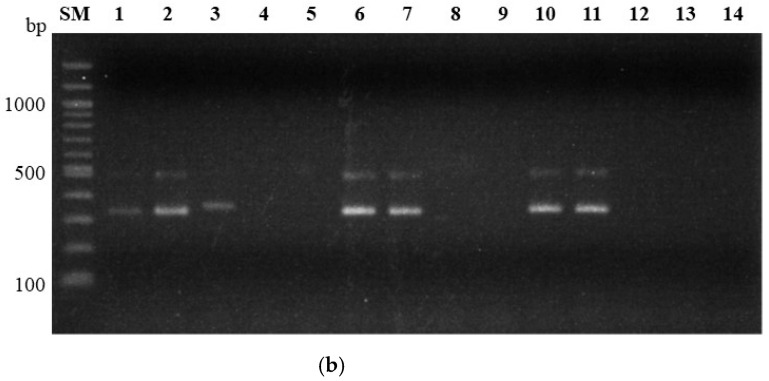
Multiplex PCR for trichothecene typing and species identification of *Fusarium* spp. Multiplex PCR with primers, 3CON, 3D3A, 3D15A, and 3NA for trichothecene typing (**a**). Expected amplicon size for 3-acetyl deoxynivalenol (3ADON): 243 bp; 15-acetyl deoxynivalenol (15ADON): 610 bp; nivalenol (NIV): 840 bp. Multiplex PCR with primers HS974, HS976, HS1035, and HS1039 for species identification (**b**). Expected amplicon size for *Fusarium graminearum* sensu stricto (s. str.): 317 bp; *Fusarium asiaticum*: 332 bp. PCR amplicons were subjected to 1% agarose gel electrophoresis. Lane SM, 100 bp size marker; lane 1, Fg0101020 (*F. graminearum* s. str./3ADON); lane 2, Fg0201201 (*F. graminearum* s. str./15ADON); lane 3, Fa0239005 (*F. asiaticum*/NIV); lane 4, NRRL26755 (*Fusarium acaciae-mearnsii*/unknown); lane 5, NRRL34207 (*F. acaciae-mearnsii*/unknown); lane 6, NRRL26916 (*Fusarium boothii*/15ADON); lane 7, NRRL29105 (*F. boothii*/15ADON); lane 8, NRRL29010 (*Fusarium meridonale*/NIV); lane 9, NRRL28721 (*F. meridonale*/NIV); lane 10, NRRL3288 (*Fusarium culmorum*/3ADON); lane 11, NRRL25475 (*F. culmorum*/3ADON); lane 12, NRRL28062 (*Fusarium pseudograminearum*/3ADON); lane 13, NRRL28334 (*F. pseudograminearum*/unknown); lane 14, NRRL13393 (*Fusarium lunulosporum*/NIV).

These multiplex PCR conditions were also applied to other members of the *Fg* complex (*Fusarium acaciae-mearnsii*, *Fusarium boothii*, and *Fusarium meridonale*) and its related species (*Fusarium lunulosporum* and *Fusarium pseudograminearum*) to investigate the applicability of PCR; although unlike our chosen species, these have never been isolated in Japan (Figure 3).

Target DNA amplification was performed in all strains tested by multiplex PCR for trichothecene typing. The identified trichothecene types matched with previously reported data [23]. Amplicons the same size as *F. graminearum* s. str. were observed in both strains of *F. boothii* using multiplex PCR for species identification. Additionally, the homologous nucleotide sequence of the amplified region of *FGSG_17538* was detected in the genome database (whole-genome shotgun contigs) of *Fusarium austroamericanum* (accession VSSV01000012.1), *F. boothii* (JAGDVE010000010.1), *Fusarium brasilicum* (JAGDVC010000008.1), *F. cortaderiae* (JAGDVB010000002.1), *Fusarium gerlachii* (JAGDUY010000005.1), *Fusarium louisianense* (JAGDUV010000021.1), *Fusarium mesoamericanum* (JAGDUS010000059.1), and *Fusarium* sp. CBS 123663 (JAGDUJ010000036.1) in the *Fg* complex with E-values from 3e^−163^ to 3e^−152^ and *F. pseudograminearum* (JTGC01000409.1), with an E-value of 1e^−144^, but not in the genome database of *F. culmorum*. A significant homologous nucleotide sequence of the amplified region in *APS1* was only found in the genome sequence of *F. asiaticum*.

**Figure 4:**
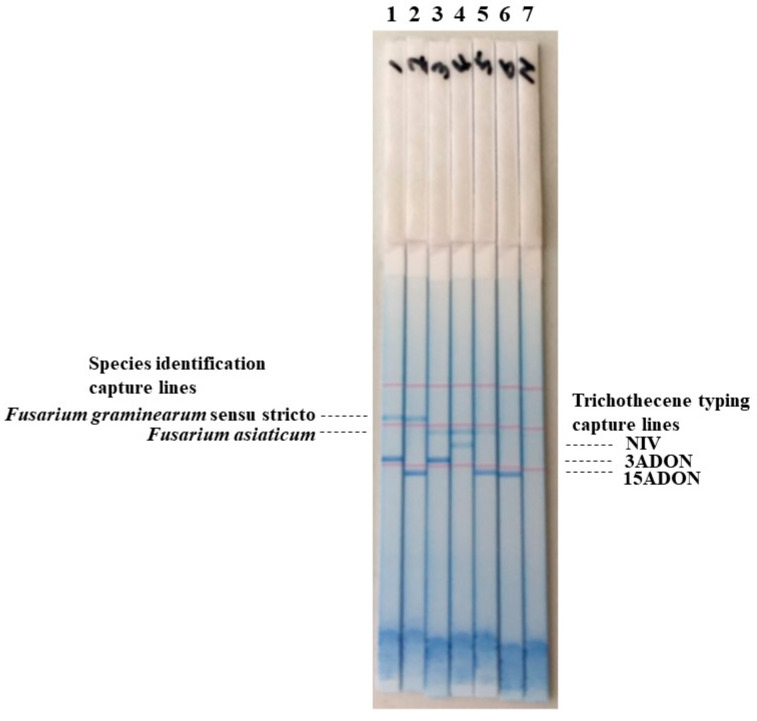
The dipstick DNA chromatography assay for species identification and trichothecene typing of the *Fusarium graminearum* species complex. Multiplex PCR solutions for species identification (*Fusarium graminearum* sensu stricto [s. str.] and *Fusarium asiaticum*) and trichothecene typing (3-acetyl deoxynivalenol, 3ADON; 15-acetyl deoxynivalenol, 15ADON; and nivalenol, NIV) were mixed and developed using dipstick DNA chromatography. Lane 1, Fg0101020 (*F. graminearum* s. str./3ADON); lane 2, Fg0201201 (*F. graminearum* s. str./15ADON); lane 3, Fa0244004 (*F. asiaticum*/3ADON); lane 4, Fa0239005 (*F. asiaticum*/NIV); lane 5, Fa0343747 (*F. asiaticum*/15ADON); lane 6, Fv0301112 (*Fusarim vorosii*/15ADON); lane 7, water control.

A dipstick DNA chromatography assay was performed using a mixture of multiplex PCR for species identification and trichothecene typing. The tested PCR mixtures included all *Fg* complex members and trichothecene types that have been detected in Japan. The PCR amplicons used for trichothecene typing were designed to be bound on the lower three lines, and PCR amplicons for species identification were designed to be bound on the two upper lines of the dipstick (Figure 4). In all the strains tested, positive lines appeared 20 min after chromatography development (Figure 4).

### 3.3. Practical Use of the FG21 Medium and the Dipstick DNA Chromatography Assay

**Figure 5:**
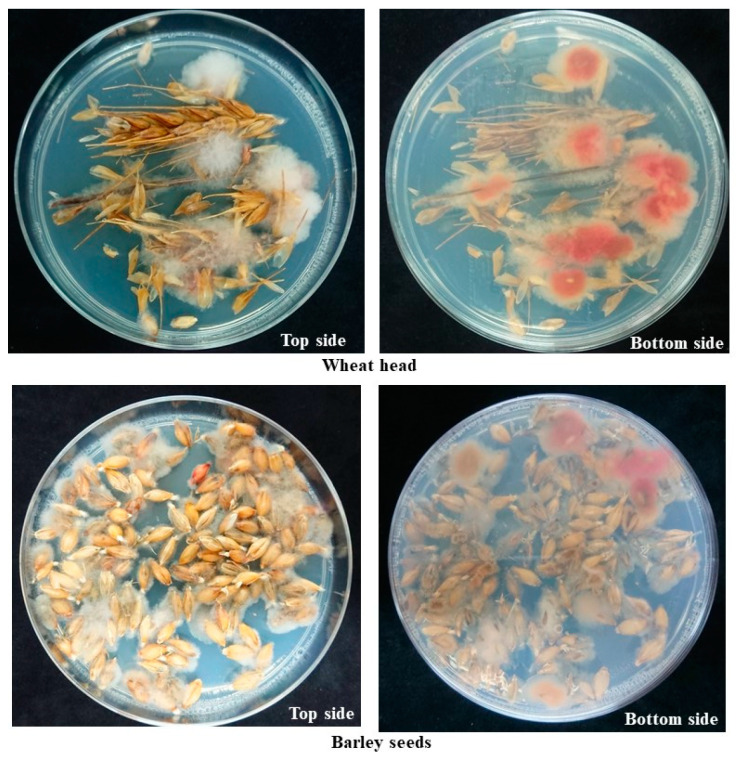
Isolation of species in the *Fusarium graminearum* species complex from wheat head or barley seeds on the FG21 medium. Wheat head or barley seeds were placed on FG21 medium after surface sterilization and kept at 25 °C for 3 days. Reddish colonies were identified as nivalenol type of *Fusarium asiaticum* using a dipstick DNA chromatography assay.

Barley seeds were collected from fields where FHB symptoms were observed in 2022. Additionally, wheat heads with FHB symptoms were collected in 2022. They were used as sources for fungal isolation using the FG21 medium. Reddish colonies that were assumed to be *Fg* complex members appeared on the FG21 medium (Figure 5).

The barley seeds yielded 4 colonies, and the wheat heads yielded 19 colonies. Each colony was obtained from barley seeds equivalent to a 30 mL volume of different fields or a wheat head of different fields. DNA was extracted from aerial mycelia and used in the dipstick DNA chromatography assay. All the 23 colonies were identified as *F. asiaticum* (Table S1). Nine colonies were identified as NIV and thirteen colonies as 3ADON (Table S1). After single colony isolation, the dipstick DNA chromatography assay was performed again. The results were similar, except for one colony, Fgc22081501. Before single-colony isolation, this colony showed positivity for NIV and 3ADON and only NIV after single-colony isolation (Table S1).

## 4. Discussion

In this study, FG21 medium was developed to be semi-selective for *Fg* complex members without requiring PCNB. In addition, a dipstick DNA chromatography assay was developed for faster and easier identification of *Fg* complex members (*F. graminearum* s. str. and *F. asiaticum*) and trichothecene typing (3ADON, 15ADON, and NIV) in Japan.

We developed the FG21 medium, owing to the lack of suitable media for isolation of *Fg* complex members. The FG medium was previously developed for *F. graminearum* isolation by modifying the Komada medium, but it used PCNB, which is now difficult to obtain [20]. PCNB is not included in the Fo-G2 medium; however, *Fg* complex members do not grow on this medium, which also contains the fungicide, iminoctadine triacetate, and the fungicide has been used for FHB control in Japan [21]. PDA was chosen as the basic medium for isolation of *Fg* complex members because the characteristic red pigmentation can be easily seen. The FG21 medium was developed for easy preparation; therefore, the medium included the minimum number of reagents. Chloramphenicol was the sole antibiotic used to inhibit bacterial growth in FG21 medium, and the pH was not adjusted. To improve bacterial growth suppression, adjusting the pH < 4 and/or supplementation with other antibiotics may be an option if bacterial growth disturbs the isolation of *Fg* complex members.

Because cryptic species have been discovered in the *Fg* complex, methods to determine species and trichothecene-type compositions have been developed in different countries [27]. In addition to biogeographical differences in the species, some species have host plant preferences: *F. asiaticum* for rice and *F. boothii* and *F. meridonale* for maize, although these preferences may be due to local conditions, such as climate, crop rotation, and presence/absence of competitors. The major FHB pathogen was *F. culmorum* before 2000 in the Netherlands, but it was replaced with *F. graminearum* s. str. in the following year [28,29]. Furthermore, the geographical alteration of trichothecene type within a species has been observed in Canada [12], the United States [11], and China [30]. These investigations demonstrate the importance of monitoring species and trichothecene-type compositions for risk assessment of members of the *Fg* complex, both as mycotoxin producers and plant pathogens.

PCR-based molecular tools to investigate species and trichothecene types have been developed for the *Fg* complex [3,31]. Niessen and Vogel [32] developed a loop-mediated isothermal amplification (LAMP) assay, based on the galactose oxidase gene (*GaoA*), to detect *Fg* complex members. The LAMP assay could uniquely detect all nine species of the *Fg* complex tested; however, each strain of *F. asiaticum* and *F. cortaderiae* were not detected. Knoll et al. [33] developed a method using DNA detection test strips to identify *F. graminearum* based on the PCR amplification of *GaoA*. Similar to the dipstick DNA chromatography assay, special instruments and agarose gel electrophoresis were not required. However, the detection target of the method was *F. graminearum* before division into 16 species, and it could not be used for species identification or trichothecene typing.

The current study demonstrated easier identification of Japanese species in the *Fg* complex using of FG21 medium and a dipstick DNA chromatography assay. The FG21 medium is convenient for mass screening of seeds, has high sensitivity, and lower investigational costs, compared to other DNA-based diagnostic procedures. Because visually assessing colonies cannot identify all species present, a dipstick DNA chromatography assay that can accurately identify two Japanese species in the *Fg* complex was developed. Fungal isolation by FG21 medium and identification by dipstick DNA chromatography assay were completed in approximately 3 days. The species of the *Fg* complex and the trichothecene type can be either simultaneously or separately identified with this assay.

A small amount of mycelia from a colony underwent DNA extraction, and two positive lines for trichothecene types were observed with the dipstick DNA chromatography assay (Table S1). This result suggests that multiple individuals may be present in a colony from the plant matter. If this possibility is eliminated, a single spore or single colony is essential prior to running the dipstick DNA chromatography assay.

*F. asiaticum* and *F. graminearum* s. str. were identified using the developed dipstick DNA chromatography assay. Because only the *F. graminearum* s. str. genome contains *FGSG_17538*, this was selected as its target gene [25]. Recently, the whole-genome sequences of all 16 species, including *Fusarium* sp. CBS 123663, was reported [34,35,36], and *FGSG_17538* or its homolog was detected in nine species, constituting a subclade in the phylogenomic tree of the *Fg* complex [36]. Furthermore, positive amplification by PCR with HS976/HS1039 primers was observed for *F. boothii*, which belongs to the African clade (Figure 3). Except for *F. graminearum* s. str., multiple *Fg* complex members with homologous *FGSG_17538* have never been isolated in Japan. Therefore, species misidentification by the dipstick DNA chromatography assay using *FGSG_17538* is unlikely to occur without new *Fg* complex member invasion in Japan. Although *F. vorosii* cannot be identified with the dipstick DNA chromatography assay, only two strains of *F. vorosii* have been detected in northern Japan. Positive amplifications for trichothecene typing were observed in *Fg* complex members, including *F. vorosii* and *Fusarium* spp., by multiplex PCR (Figure 3). However, DNA amplification for species identification was negative for *F. vorosii* and *Fusarium* spp. Therefore, *F. vorosii* and *Fusarium* spp. cannot be discriminated by the dipstick DNA chromatography assay currently. However, if genes that are uniquely present in a single species can be discovered, such as *APS1* for *F. asiaticum*, these genes can be targeted to identify all species in the *Fg* complex.

The species and trichothecene-type composition of the *Fg* complex have been investigated in countries worldwide. Faster and easier molecular diagnostic tools are useful for monitoring the dynamic changes in *Fg* complex members.

## 5. Patents

Application number for the dipstick DNA chromatography assay is (2020) 27892.

## Figures and Tables

**Table 1:** Strains used in the current study.

Species	Strain ^a^	Application	Trichothecene Type ^b^	Reference
*Fusarium graminearum* sensu stricto	Fg0101020	FG21 medium, Multiplex PCR, dipstick DNA chromatography assay	3ADON	[9]
	Fg0201201	FG21 medium, Multiplex PCR, dipstick DNA chromatography assay	15ADON	[9]
*Fusarium asiaticum*	Fa0244004	FG21 medium, Multiplex PCR, dipstick DNA chromatography assay	3ADON	[9]
	Fa0239005	FG21 medium, Multiplex PCR, dipstick DNA chromatography assay	NIV	[9]
	Fa0343747	FG21 medium, Multiplex PCR, dipstick DNA chromatography assay	15ADON	[9]
*Fusarium vorosii*	Fv0301112 ^c^	FG21 medium, Multiplex PCR, dipstick DNA chromatography assay	15ADON	[3,9]
	Fv0301831 ^d^	FG21 medium		
*Fusarium acaciae-mearnsii*	NRRL26755	Multiplex PCR	NIV *	
	NRRL34207	Multiplex PCR	NIV *	
*Fusarium boothii*	NRRL26916	Multiplex PCR	15ADON	[23]
	NRRL29105	Multiplex PCR	15ADON	[23]
*Fusarium meridonale*	NRRL29010	Multiplex PCR	NIV	[23]
	NRRL28721	Multiplex PCR	NIV	[23]
*Fusarium cerealis*	NRRL25805	FG21 medium		
*Fusarium culmorum*	MAFF101144	FG21 medium		
	NRRL3288	FG21 medium, Multiplex PCR	3ADON	[23]
	NRRL25475	Multiplex PCR	3ADON	[23]
*Fusarium fujikuroi*	Gfc0801001	FG21 medium		[24,25]
	Gfc0825009	FG21 medium		[24,25]
*Fusarium lunulosporum*	NRRL13393	Multiplex PCR	NIV	[23]
*Fusarium poae*	MAFF236500	FG21 medium		
	MAFF236648	FG21 medium		
*Fusarium pseudograminearum*	NRRL28062	Multiplex PCR	3ADON	[23]
	NRRL28334	Multiplex PCR	3ADON *	
*Fusarium sporotrichioides*	MAFF236638	FG21 medium		
	MAFF236639	FG21 medium		
*Microdochium nivale*	MAFF235834	FG21 medium		
	MAFF236681	FG21 medium		
*Fusarium* sp. ^e^	F1904008	FG21 medium		
*Pestalotiopsis* sp. ^e^	P1904014	FG21 medium		
*Alternaria* sp. ^e^	A1904015	FG21 medium		
*Epicoccum* sp. ^e^	E1904019	FG21 medium		

^a^ MAFF: The Research Center of Genetic Resources, National Agriculture and Food Research Organization (NARO), Tsukuba, Japan; NRRL: Agriculture Research Service Culture Collection, National Center for Agricultural Utilization Research, USDA/ARS, Peoria, IL, United States. ^b^ * shows that trichothecene type (15-acetyl deoxynivalenol: 15ADON; 3-acetyl deoxynivalenol: 3ADON; and nivalenol: NIV) was newly identified by multiplex PCR in this study. Other parameters have been previously determined in the literature. ^c^ Equivalent strain names are NRRL38207 and MAFF241221. ^d^ Equivalent strain name is MAFF241222. ^e^ The genus was identified by a homological search of the nucleotide sequences of the internal transcribed spacer region of the nuclear ribosomal DNA.

**Table 2:** Primers used in the current study.

Application	Primer	5′- -3′	Target Gene	Function	Expected Amplification Size (bp)	Reference
Multiplex PCR for trichothecene typing	3CON	Biotin-TGGCAAAGACTGGTTCAC	*Tri3*	Reverse for all three trichothecene types		[3]
	3D15A	TagX-ACTGACCCAAGCTGCCATC	*Tri3*	15-acetyl deoxynivalenol (15ADON) type specific forward	610	[3]
	3D3A	TagY-CGCATTGGCTAACACATG	*Tri3*	3-acetyl deoxynivalenol (3ADON) type specific forward	243	[3]
	3NA	TagZ-GTGCACAGAATATACGAGC	*Tri3*	Nivalenol (NIV) type specific forward	840	[3]
Multiplex PCR for species identification	HS974	TagV-GCTGGCATGGTTGATATGGG	*APS1*	*Fusarium asiaticum* specific forward	332	
	HS1035	Biotin-GTGGGCCCGTACATGTTGA	*APS1*	*F. asiaticum* specific reverse		
	HS976	TagW-CAAGTCAAGTCTTCCACCGT	*FGSG_17538*	*Fusarium graminearum* sensu stricto specific forward	317	
	HS1039	Biotin-GCACCCAGGCCATATTCAGT	*FGSG_17538*	*F. graminearum* sensu stricto specific reverse		

## Data Availability

Not applicable.

## Supplementary Materials

The following supporting information can be downloaded at: https://www.mdpi.com/article/10.3390/jof8101048/s1, Table S1: Colonies isolated from plant source in the field.

## References

[1] Johnson D.D., Flaskerud G.K., Taylor R.D., Satyanarayana V., Leonard K.J., Bushnell W.R. (2003). Quantifying economic impacts of Fusarium head blight in wheat. Fusarium Head Blight of Wheat and Barley.

[2] O’Donnell K., Ward T.J., Geiser D.M., Kistler H.C., Aoki T. (2004). Genealogical concordance between the mating type locus and seven other nuclear genes supports formal recognition of nine phylogenetically distinct species within the *Fusarium graminearum* clade. Fungal Genet. Biol..

[3] Starkey D.E., Ward T.J., Aoki T., Gale L.R., Kistler H.C., Geiser D.M., Suga H., Tóth B., Varga J., O’Donnell K. (2007). Global molecular surveillance reveals novel Fusarium head blight species and trichothecene toxin diversity. Fungal Genet. Biol..

[4] O’Donnell K., Ward T.J., Aberra D., Kistler H.C., Aoki T., Orwig N., Kimura M., Bjørnstad A., Klemsdal S.S. (2008). Multilocus genotyping and molecular phylogenetics resolve a novel head blight pathogen within the *Fusarium graminearum* species complex from Ethiopia. Fungal Genet. Biol..

[5] Yli-Mattila T., Gagkaeva T., Ward T.J., Aoki T., Kistler H.C., O’Donnell K. (2009). A novel Asian clade within the *Fusarium graminearum* species complex includes a newly discovered cereal head blight pathogen from the Russian Far East. Mycologia.

[6] Sarver B.A.J., Ward T.J., Gale L.R., Broz K., Kistler H.C., Aoki T., Nicholson P., Carter J., O’Donnell K. (2011). Novel Fusarium head blight pathogens from Nepal and Louisiana revealed by multilocus genealogical concordance. Fungal Genet. Biol..

[7] Boutigny A.-L., Ward T.J., Van Coller G.J., Flett B., Lamprecht S.C., O’Donnell K., Viljoen A. (2011). Analysis of the *Fusarium graminearum* species complex from wheat, barley and maize in South Africa provides evidence of species-specific differences in host preference. Fungal Genet. Biol..

[8] Lee J., Chang I.Y., Kim H., Yun S.H., Leslie J.F., Lee Y.W. (2009). Genetic diversity and fitness of *Fusarium graminearum* populations from rice in Korea. Appl. Environ. Microbiol..

[9] Suga H., Karugia G.W., Ward T., Gale L.R., Tomimura K., Nakajima T., Miyasaka A., Koizumi S., Kageyama K., Hyakumachi M. (2008). Molecular characterization of the *Fusarium graminearum* species complex in Japan. Phytopathology.

[10] Feinberg B., McLaughlin C.S., Beasley V.R. (1989). Biochemical mechanism of action of trichothecene mycotoxins. Trichothecene Mycotoxicoses: Pathophysiologic Effects.

[11] Gale L.R., Harrison S.A., Ward T.J., O’Donnell K., Milus E.A., Gale S.W., Kistler H.C. (2011). Nivalenol-type populations of *Fusarium graminearum* and *F. asiaticum* are prevalent on wheat in southern Louisiana. Phytopathology.

[12] Ward T., Clear R., Rooney A., O’Donnell K., Gaba D., Patrick S., Starkey D., Gilbert J., Geiser D., Nowicki T. (2008). An adaptive evolutionary shift in Fusarium head blight pathogen populations is driving the rapid spread of more toxigenic *Fusarium graminearum* in North America. Fungal Genet. Biol..

[13] Suzuki F., Koba A., Nakajima T. (2010). Simultaneous identification of species and trichothecene chemotypes of *Fusarium asiaticum* and *F. graminearum* sensu stricto by multiplex PCR. J. Gen. Plant Pathol..

[14] Hayashi M., Natori T., Kubota-Hayashi S., Miyata M., Ohkusu K., Kawamoto K., Kurazono H., Makino S., Ezaki T. (2013). A new protocol to detect multiple foodborne pathogens with PCR dipstick DNA chromatography after a six-hour enrichment culture in a broad-range food pathogen enrichment broth. BioMed Res. Int..

[15] Nagai S., Miyamoto S., Ino K., Tajimi S., Nishi H., Tomono J. (2016). Easy detection of multiple *Alexandrium* species using DNA chromatography chip. Harmful Algae.

[16] Komada H. (1975). Development of a selective medium for quantitative isolation of *Fusarium oxysporum* from natural soil. Rev. Plant Prot. Res..

[17] Nishimura N. (2007). Selective media for *Fusarium oxysporum*. J. Gen. Plant Pathol..

[18] Edel-Hermann V., Gautheron N., Mounier A., Steinberg C. (2015). *Fusarium* diversity in soil using a specific molecular approach and a cultural approach. J. Microbiol. Methods.

[19] Rodríguez-Molina M.C., Tello-Marquina J.C., Torres-Vila L.M., Bielza-Lino P. (2000). Micro-scale systematic sampling of soil: Heterogeneity in populations of *Fusarium oxysporum*, *F. solani*, *F. roseum* and *F. moniliforme*. J. Phytopathol..

[20] Togawa M. (1994). Selective medium for isolation of *Fusarium graminearum*. Soil Microorg..

[21] Souma J., Kozawa T. (2006). Chemical control of Fusarium head blight in wheat in Hokkaido. Mycotoxins.

[22] White T.J., Bruns T., Lee S., Taylor J.W., Innis M.A., Gelfand D.H., Sninsky J.J., White T.J. (1990). Amplification and direct sequencing of fungal ribosomal RNA genes for phylogenetics. PCR Protocols: A Guide to Methods and Applications.

[23] Ward T.J., Bielawski J.P., Kistler H.C., Sullivan E., O’Donnell K. (2002). Ancestral polymorphism and adaptive evolution in the trichothecene mycotoxin gene cluster of phytopathogenic *Fusarium*. Proc. Natl. Acad. Sci. USA.

[24] Suga H., Kitajima M., Nagumo R., Tsukiboshi T., Uegaki R., Nakajima T., Kushiro M., Nakagawa H., Shimizu M., Kageyama K. (2014). A single nucleotide polymorphism in the translation elongation factor 1α gene correlates with the ability to produce fumonisin in Japanese *Fusarium fujikuroi*. Fungal Biol..

[25] Suga H., Arai M., Fukasawa E., Motohashi K., Nakagawa H., Tateishi H., Fuji S.-I., Shimizu M., Kageyama K., Hyakumachi M. (2019). Genetic differentiation associated with fumonisin and gibberellin production in Japanese *Fusarium fujikuroi*. Appl. Environ. Microbiol..

[26] Walkowiak S., Rowland O., Rodrigue N., Subramaniam R. (2016). Whole genome sequencing and comparative genomics of closely related Fusarium head blight fungi: *Fusarium graminearum*, *F. meridionale* and *F. asiaticum*. BMC Genom..

[27] Van der Lee T., Zhang H., van Diepeningen A., Waalwijk C. (2015). Biogeography of *Fusarium graminearum* species complex and chemotypes: A review. Food Addit. Contam. Part A Chem. Anal. Control Expo. Risk Assess..

[28] De Nijs M., Larsen J.S., Gams W., Rombouts F.M., Wernars K., Thrane U., Notermans S.H.W. (1997). Variations in random amplified polymorphic DNA patterns and secondary metabolite profiles within *Fusarium* species from cereals from various parts of the Netherlands. Food Microbiol..

[29] Waalwijk C., Kastelein P., De Vries I., Kerényi Z., van der Lee T., Hesselink T., Köhl J., Kema G. (2003). Major change in *Fusarium* spp. in wheat in the Netherlands. Eur. J. Plant Pathol..

[30] Zhang H., van der Lee T., Waalwijk C., Chen W., Xu J., Xu J., Zhang Y., Feng J. (2012). Population analysis of the *Fusarium graminearum* species complex from wheat in China show a shift to more aggressive isolates. PLoS ONE.

[31] Jennings P., Coates M.E., Walsh J.A., Nicholson P. (2004). Determination of deoxynivalenol- and nivalenol-producing chemotypes of *Fusarium graminearum* isolates from wheat crops in England and Wales. Plant Pathol..

[32] Niessen L., Vogel R.F. (2010). Detection of *Fusarium graminearum* DNA using a loop-mediated isothermal amplification (LAMP) assay. Int. J. Food Microbiol..

[33] Knoll S., Vogel R.F., Niessen L. (2002). Identification of *Fusarium graminearum* in cereal samples by DNA detection test strips^TM^. Lett. Appl. Microbiol..

[34] Cuomo C.A., Guldener U., Xu J.-R., Trail F., Turgeon B.G., Di Pietro A., Walton J.D., Ma L.-J., Baker S.E., Rep M. (2007). The *Fusarium graminearum* genome reveals a link between localized polymorphism and pathogen specialization. Science.

[35] Kelly A.C., Ward T.J. (2018). Population genomics of *Fusarium graminearum* reveals signatures of divergent evolution within a major cereal pathogen. PLoS ONE.

[36] Kulik T., Molcan T., Fiedorowicz G., van Diepeningen A., Stakheev A., Treder K., Olszewski J., Bilska K., Beyer M., Pasquali M. (2022). Whole-genome single nucleotide polymorphism analysis for typing the pandemic pathogen *Fusarium graminearum* sensu stricto. Front. Microbiol..

