# Supplementary material for: A Novel Medium for Isolating Two Japanese Species in the Fusarium graminearum Species Complex and a Dipstick DNA Chromatography Assay for Species Identification and Trichothecene Typing
Source: J Fungi (Basel). 2022 Oct 5;8(10):1048. doi: 10.3390/jof8101048 (PMC9605519; doi:10.3390/jof8101048)
Supplement: Supplementary file 1 [file jof-08-01048-s001.zip › jof-1915987-supplementary.pdf]

Table S1. Colonies isolated from plant source in the field.

Isolation Source				Harvested Year	Location
Barley seeds[c]	2022	Ibaraki prefecture	Colony[a]	Species[b]	Trichothecene type[b]
			Fgc22081301	*Fusarium asiaticum*	NIV
			Fgc22081401	*Fusarium asiaticum*	NIV
			Fgc22081501	*Fusarium asiaticum*	NIV and 3ADON[d]
			Fgc22081601	*Fusarium asiaticum*	NIV
Wheat head[e]	2022	Mie prefecture	Fgc22241701	*Fusarium asiaticum*	NIV
			Fgc22241801	*Fusarium asiaticum*	3ADON
			Fgc22241901	*Fusarium asiaticum*	3ADON
			Fgc22242001	*Fusarium asiaticum*	3ADON
			Fgc22242101	*Fusarium asiaticum*	NIV
			Fgc22242201	*Fusarium asiaticum*	3ADON
			Fgc22242301	*Fusarium asiaticum*	3ADON
			Fgc22242401	*Fusarium asiaticum*	3ADON
			Fgc22242501	*Fusarium asiaticum*	3ADON
			Fgc22242601	*Fusarium asiaticum*	3ADON
			Fgc22242701	*Fusarium asiaticum*	NIV
			Fgc22242801	*Fusarium asiaticum*	3ADON
			Fgc22242901	*Fusarium asiaticum*	NIV
			Fgc22243001	*Fusarium asiaticum*	3ADON
			Fgc22243101	*Fusarium asiaticum*	3ADON
			Fgc22243201	*Fusarium asiaticum*	NIV
			Fgc22243301	*Fusarium asiaticum*	3ADON
			Fgc22243401	*Fusarium asiaticum*	3ADON
			Fgc22243501	*Fusarium asiaticum*	NIV

[a] Each colony was obtained from barley seeds equivalent to 30 mL volume from different fields or a wheat head of different fields with the FG21 medium.

[b] Species (*Fusarium asiaticum* and *Fusarium graminearum* sensu stricto) and trichothecene types (15-acetyl deoxynivalenol: 15ADON; 3-acetyl deoxynivalenol: 3ADON; and nivalenol: NIV) were identified by dipstick DNA chromatography. The dipstick DNA chromatography assay was performed for sample before and after single colony isolation, and obtained results were the same for all colonies but Fgc22081501.

[c] Seeds were collected from fields where Fusarium head blight symptoms were observed.

[d] The dipstick DNA chromatography assay showed a positive line for NIV and 3ADON with the sample before single-colony isolation. The dipstick DNA chromatography assay with the sample after single-colony isolation showed a positive line only for NIV.

[e] Wheat head with Fusarium head blight symptoms.
